# Effect of ewe’s (semi-skimmed and whole) and cow’s milk yogurt consumption on the lipid profile of control subjects: a crossover study

**DOI:** 10.1080/16546628.2017.1391669

**Published:** 2017-10-26

**Authors:** Begoña Olmedilla-Alonso, Esther Nova-Rebato, Natalia García-González, Ana-Belén Martín-Diana, Javier Fontecha, David Delgado, Ana-Elisa Gredilla, Francisco Bueno, Carmen Asensio-Vegas

**Affiliations:** ^a^ Department of Metabolism and Nutrition, Institute of Food Science, Technology and Nutrition (ICTAN-CSIC), Madrid, Spain; ^b^ Agro Technological Institute, Deputy Directorate of Research and Technology, Dairy Technological Station, Palencia, Spain; ^c^ Agro Technological Institute, Deputy Directorate of Research and Technology, Area of Innovation and Process Optimisation, Valladolid, Spain; ^d^ Department of Bioactivity and Food Analysis, Group of Lipids, Research Institute of Food Science (CIAL; CSIC-UAM), Madrid, Spain

**Keywords:** dairy products, lipid profile, intervention study, HDL-cholesterol, LDL-cholesterol, cardiovascular disease markers

## Abstract

Yogurt is the most widely consumed fermented milk product worldwide. Studies have mainly used milk and dairy products from cow, which have a lower fat content than those from ewe and a different lipid profile. This study investigated the effect on the lipid profile of control subjects of three different set yogurts: (a) semi-skimmed ewe´s milk yogurt (2.8% milk fat); (b) whole ewe´s milk yogurt (5.8 % milk fat); (c) cow´s milk yogurt (3 % milk fat). A randomized crossover study included 30 healthy adults (16 women) to consume 250 g/yogurt/day during three consecutive 5-weeks periods separated by 4-week washouts. Blood samples were collected at the start and end of each period for the analysis of serum cholesterol (total, HDL-, LDL-) and triglycerides. We found no differences in the serum concentrations of lipid and lipoprotein fractions of the volunteers after the intake of any of the three types of yogurts. When the volunteers were grouped into two risk groups of risk according to their total cholesterol/HDL cholesterol ratio, the same differences between the groups in terms of the cholesterol (HDL-, LDL-) and triglyceride responses at baseline and after yogurt intake were found, with no effects due to the different types of yogurts. Moreover, we performed compositional analysis of the yogurts including determination of protein, fat, minerals and fatty acids (FA). Contents in protein, calcium, magnesium, non-protein nitrogen and some FA (mainly short-chain-FA) were higher for ewe’s than for cow’s milk yogurt. n6-n3 ratio was lower in the ewe’s milk yogurt. In conclusion, yogurt intake, from ewe’s and cow’s milk, at levels of consumption compatible with a varied diet, neither decreases nor increases plasma lipoprotein cholesterol levels in apparently healthy individuals. As ewe’s milk yogurt has a high content of macro- and micronutrients, certain target populations could benefit from its consumption.

## Introduction

Fermented milk beverage consumption is on the rise due to consumers’ perception of its healthy effects, widely disseminated by increasing numbers of studies describing the importance of the different nutrients and bioactive compounds. The consumption of yogurt is also growing, with the consequent decrease in the consumption of liquid milk, especially associated with intolerance to lactose and the increase in fermented products like Greek-style yogurt [1,2]. According to the FAO (2015), 85% of the world milk production is derived from cattle, followed by milks from other species such as buffalo (11%), goat (2.3%), sheep (1.4%), and camel (0.2%) [3], and milk is processed for 3500 different fermented foods worldwide [4]. However, dairy sheep farms represent a significant part of agrarian economies in many countries, especially those bordering the Mediterranean Sea and in the Middle East [5–8]. In Spain, the production of ewe’s milk represents 8% of the total (from cow, ewe and goat), most of which comes from the central region (Castilla y León) (69.5%) [9]. It is used mainly in making farmhouse cheese because of its high fat and protein content [10] and the organoleptic characteristics of the final product.

Yogurt is the form in which the major part of the fermented milk utilized in Spain is consumed, representing 84.9% of the total fermented milk production [11] to satisfy a yogurt consumption of 9.7 L/p/y [12]. The properties of yogurt (level of acidity, fatty acid [FA] composition and aromatic compounds), its nutritional value, and its sensorial profile are influenced by the composition of the milk used to prepare it, as by the processing conditions and the activity of the inocula during fermentation [13,14]. Ewe’s milk is especially suitable for making yogurt, owing to its high levels of protein and total solid content, with respect to cow’s milk, and higher content in minerals, vitamins, and fat [15]. Thus, ewe’s milk confers a different texture in yogurt compared with those prepared with cow’s milk, making it creamier and giving it an increased consistency that favours freezing without phase separation [8].

Ewe’s milk has a higher fat content than cow’s milk; in addition, the lipid profiles of the two milks differ significantly, with higher concentrations of short- and medium FA in the former [15–18]. The polyunsaturated fatty acids (PUFAs) in sheep milk fat comprise linoleic (cis-9, cis-12 C18:2) and α-linolenic (cis-9, cis-12, cis-15 C18:3) acids, as well as smaller concentrations of their isomers [19]. Mono- and polyunsaturated FA in sheep milk may contribute to the prevention of cardiovascular diseases owing to their atherogenic and thrombogenic indixes [20,21]. Among ruminants, sheep milk fat contains not only one of the highest levels of conjugated linoleic acid (0.65 g CLA/100 g of FA) but also a large amount of vaccenic acid, its physiological precursor [22]. The CLA isomers that appear in larger quantities and have a beneficial functional value are the cis-9, trans-11 CLA, and trans-10, cis-12 CLA with anticarcinogenic and lipolytic activities (weight loss effect), respectively [23–26]. Short-chain FA have been seen to have no detrimental effect on cholesterolemia, and some have even shown a beneficial effect on cholesterol metabolism [27], are benign with regard to inflammation, and might actually be beneficial to some population segments [28]. On the other hand, agglutinin is absent from sheep and goat milk, providing better digestibility than cow’s milk [24].

Although milk and dairy products are important sources of macro- and micronutrients in the human diet [20,29], the relatively high saturated fats (SF) raise issues of potential detrimental effects, namely on the cardiovascular system [31]. However, the majority of studies in recent years suggest no adverse effects on surrogate markers of cardiovascular disease (CVD) or cardiovascular prognosis [18,32]. The milk and dairy products used in those studies are mostly from cow and have a lower fat content than those from ewe’s milk, and their lipid profile is different. Thus, studies must be developed to assess the effect of the consumption of dairy products made from milks of animals other than the cow, during an intervention period of three weeks as the minimum needed to evaluate the effect of SF consumption on serum LDL-cholesterol concentration [33].

The aim of this study was to compare the effects of yogurts from ewe’s milk (whole and semi- skimmed, 5.8% and 2.8% fat, respectively) and cow’s milk (3% fat) in a randomized, crossover intervention study in apparently healthy subjects, evaluating lipid profile (total cholesterol, HDL- and LDL-cholesterol, triglycerides) as coronary heart disease (CHD) risk biomarkers.

## Material and methods

### Study design

**Figure 1.**
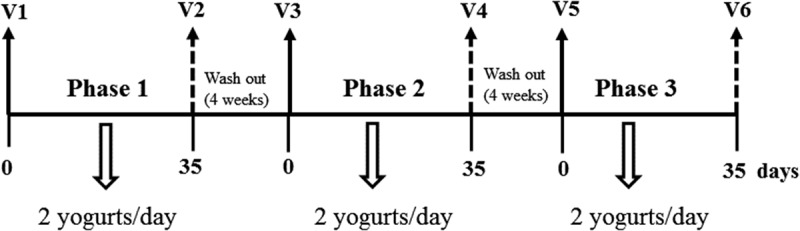
Study design. Blood samples and dietary records collected at each time point (V1 to V6).

We performed a randomized, controlled, crossover study of 6 month’s duration. Participants consumed two yogurts per day (125 g/yogurt) during each of the three 5-week study phases, followed by a 4-week washout interval (Figure 1). Every period lasted long enough to produce significant changes in the lipid profile and the lipoprotein levels [34], as well as to return to the baseline lipid profile after the washout periods [35,36].

During the three phases of the study, the volunteers consumed in random order the three types of yogurts: whole ewe’s milk yogurt (5.8% milk fat), semi-skimmed ewe’s milk yogurt (2.8% milk fat) and whole cow’s milk yogurt (3.0% milk fat). The yogurts, freshly made, were delivered to the volunteers twice during each of the periods. During the washout periods, the subjects followed their habitual diets, avoiding eating probiotic products or probiotic yogurts.

Blood samples were collected after an overnight fast (at least 9 hours) at the start and end of each intervention period from all participants, and serum obtained for the analysis of the cholesterol (total, HDL-cholesterol, and LDL-cholesterol) and triglycerides, as CHD biomarkers to assess the impact of the regular consumption of ewe’s milk yogurts with different fat contents and profiles from those made with the cow’s milk most commonly available in the market. Serum was separated from blood by centrifugation at 2,500 *g* 15 min and kept frozen at –80°C until analysis. Subjects completed 3-day dietary records at the beginning of each phase in order to calculate their dietary nutrient intake. There were no differences in the dietary macronutrient intake in any of the study periods (data not shown).

### Ethics statement

The study was approved by the Ethics Committee for Clinical Research of Hospital Universitario Puerta de Hierro-Majadahonda (Record no. 305, dated 9 December 2014) and the Bioethics Committee of the Spanish Scientific Research Council (CSIC). All subjects gave their written informed consent after receiving oral and written information about the study. The study was carried out in accordance with the Declaration of Helsinki. After completion of all measurements, participants were financially compensated (200 euros).

### Subjects

Thirty volunteers (14 men, 16 women) were enrolled in a crossover study and all of them completed the study. Participants were selected from 64 individuals who were interested and contacted through advertisements. Selected participants met the inclusion criteria: serum cholesterol 4.65–6.20 mmol/L, normal or overweight (BMI within the range 19–28 kg/m^2^), mixed diet (no avoidance of any food groups), and willingness to consume two yogurts per day. The exclusion criteria were as follows: use of drugs or phytosterol-enriched beverages/foods to control cholesterol levels, hypertension and/or obesity, use of anti-inflammatory medication, and chronic diseases that can affect lipid metabolism (i.e. diabetes, cardiovascular disease).

### Blood samples

Total cholesterol, triglycerides, HDL-cholesterol, and LDL-cholesterol were measured in serum samples (ADVIA Chemistry 2400 of Siemens®). We measured triglycerides by GPO Trinder method without serum reference, a colorimetric and enzymatic method in three steps [37]. HDL-cholesterol was measured by means of the removing/catalase enzymatic method. Total cholesterol was measured by an enzymatic method mediated by cholesterol-esterase and cholesterol-oxidase followed by a Trinder reaction. The low-density lipoprotein (LDL) cholesterol level was calculated with the Friedewald equation [38].

Different indexes were also calculated as CHD biomarkers: the atherogenic index (total cholesterol/HDL-cholesterol) and the LDL-cholesterol/HDL-cholesterol ratio [39].

### Yogurts

Yogurt was prepared using fresh raw ewe’s milk from *Churra* sp. (traditional breed in Castilla y León, Spain) provided by the School of Viñalta (Palencia, Spain), where the animals were handled in accordance with Directive 2010/63/EU [40] for the protection of animals used for scientific purposes. Cow’s milk yogurt used in this study was from a commercial brand with a high market share in Spain.

For ewe’s yogurt we used a commercial yogurt culture, YF-L903, which contains a mixture of *Streptococcus thermophilus* and *Lactobacillus bulgaricus* [41]. Starters used for direct vat inoculation were provided by Chr. Hansen, Madrid, Spain.

The milk was processed in Estación Tecnológica de la Leche (ETL, Palencia), in northern Spain, within 2 hours after reception. Milk base was strained using a cloth filter and divided into two batches of 30 L, and ewe’s milk was previously standardized and pasteurized at 80°C for 30 min. A portion of the ewe’s milk from each batch was subjected to a skimming process. Then, the milk was cooled to 42–43°C and inoculated with starters. The lyophilized cultures were prepared individually according to commercial recommendations. Each spray-dried culture (50 U 250 L^−1^) was dissolved in 1 L of ewe’s milk at 42°C, and 4 mL L^−1^ of each started culture was inoculated into the corresponding batch. Both mixtures were transferred to tightly closed plastic cups (120 mL, Alta Barrera SL, Barcelona, Spain) and incubated (Portinox ARGBT 700-1P, Sevilla, Spain) at 42°C until the pH reached 4.6. Then, samples were stored at 4°C and sent to Institute of Food Science, Technology and Nutrition (ICTAN) (Madrid) to be used in the intervention study. Duplicate aliquots of each sample were taken and subjected to physicochemical and microbiological analyses.

### Physicochemical properties of yogurts

In recently prepared yogurt (day 1) we measured lactose [42] and pH. We took 2 g of the yogurt samples and homogenized them in 8 mL of distilled water. The pH was measured at room temperature (20°C ± 2°C) using a pH meter (model pH-MATIC 23, Crison, Ltd, Barcelona, Spain). The following analyses were also carried out on day 1, according to ISO procedures: fat [43], protein [44], and total solid content [45]; Ca, Mg, and K were quantified using an Ion Chromatograph 882 Compact IC Plus (Metrohm AG, Herisau, Switzerland) with a conductivity detector.

Microbiological analysis of the yogurts was performed by plating serial dilutions of ewe’s fermented milk with the starter at day 7 of storage. Twenty grams of yogurt sample was decimally diluted in sterile peptone water (0.1% w v^−1^). After uniform mixing using an IUL Masticator BASIC (IUL S.A., Barcelona, Spain), serial decimal dilutions were prepared in 9 ml of sterile peptone water with subsequent inoculation of 1 ml of each dilution into two Petri dishes. The selective enumeration of *Streptococcus thermophilu*s sp. was performed using the pour plate method on M17 agar (Oxoid, Basingstoke, UK) (incubated aerobically at 37°C for 48 h), whereas *Lactobacillus bulgaricus* sp. was enumerated on acidified (pH 5.4 with glacial acetic acid) MRS agar (Difco, US) (microaerophilic conditions at 37°C for 72 h). *S. thermophilus* sp. and *L. bulgaricus* sp. counts were quantified after incubation according to a standardized method [46].

### Fatty acid analysis in ewe’s and cow’s milk yogurts by GC-MS

Yogurt fat was extracted from the freeze-dried yogurt samples following the Folch method modified according to Castro-Gómez et al. [47]. The fat samples were derivatized following the method described in Castro-Gómez et al. [48]. Fatty acid methyl esters (FAMEs) were analysed using a CP-Sil 88 fused-silica capillary column (100 m × 0.25 mm ID × 0.2 micron, Chrompack, Middelburg, The Netherlands) in an Agilent chromatograph (Agilent Technologies, Inc., Wilmington, USA) (model 6890N) fitted with an MS detector (Agilent 5973N) that was operated in the scan mode from 50 to 550 Da. Chromatographic conditions were as in Rodríguez-Alcalá and Fontecha [49].

### Statistical analysis

Sample size calculation was performed on the basis of a mean value for baseline LDL-cholesterol of 3.23 mmol/L (SD 0.52 mmol/L). A sample size of 29 subjects is necessary to obtain a 10% difference in the LDL-cholesterol with 90% power and an alpha error of 0.05. Results are expressed as the mean and standard deviation. All data showed a normal distribution, assessed using a normal probability plot and the Kolmogorov–Smirnov test.

There were no statistical differences between the mean baseline levels of the three phases of the study (mixed general linear model [GLM], with ‘yogurt variety’ as a fixed factor and ‘visit’ as a random factor). A GLM followed by the Bonferroni test was used to assess the statistical differences between periods for each parameter in terms of relative percent changes. Since there was some variability among participants owing to the individual cardiovascular risk (total cholesterol/HDL-cholesterol index), the top tertile, including the six women and four men with the highest values for this index before the intervention was started, was compared with the remaining 20 individuals (the intermediate and low tertiles together). The cut-off points for the highest tertile were ≥5.1 for men and ≥3.5 for women. All the statistical analyses were performed using IBM SPSS Statistics 22.0 (Armonk, NY; IBM Corp.)

## Results

The baseline characteristics of the subjects at the initiation of the first intervention period of the study are listed in Table 1. The participants showed normal cholesterol (under 5.17 mmol/L) or borderline high cholesterol (5.17–6.18 mmol/L, and 3.36–4.11 mmol/L LDL-cholesterol) and an average BMI of 25.1 kg/m^2^.

**Table 1.** Baseline characteristics of the volunteers (*n* = 30; 14 men and 16 women) at the start of the intervention study (mean ± SD).

	Baseline levels	
	Men (*n* = 14)	Women (*n* = 16)
Age (years)	43 ± 13	41 ± 14
Weight (kg)	82.7 ± 12.1	60.7 ± 8.3
BMI (kg/m^2^)	26.8 ± 2.8	23.6 ± 2.8
Total cholesterol (mmol/L)	4.91 ± 0.65	5.39 ± 0.75
HDL-cholesterol (mmol/L)	1.24 ± 0.22	1.74 ± 0.25
LDL-cholesterol (mmol/L)	3.36 ± 0.61	3.41 ± 0.67
Triglycerides (mmol/L)	1.22 ± 0.48	0.94 ± 0.34
Total cholesterol/HDL-cholesterol ratio	4.08 ± 0.97	3.14 ± 0.49
Systolic blood pressure (mm Hg)	130 ± 19	112 ± 13
Diastolic blood pressure (mm Hg)	83 ± 12	74 ± 9

Table 2 shows some nutritional information, FA profile, and atherogenic index (AI) in the ewe’s milk yogurts used in this study and of the commercial cow’s milk yogurt. The two types of ewe’s milk yogurt differed in fat content (approx. 6% in whole vs approx. 2.9% in semi-skimmed ewe’s milk yogurt; approx. 2.9% in whole cow’s milk yogurt), but the relative composition of FA (g/100 g fat) was maintained. The saturated fatty acids (SFA) were significantly higher in ewe’s than in cow’s yogurts, and the short-chain FA were almost twofold higher in ewe’s than in cow’s yogurt (*p *< 0.05). Although there were no significant differences in the PUFA or CLA content between yogurt samples, the ewe’s milk yogurt presented almost double the n-3 alpha linolenic acid than cow’s milk and had a lower n-6:n-3 ratio. The protein content was twice as much in ewe as in cow yogurts. Calcium, magnesium, and nitrogen non-protein contents were higher in ewe yogurts than in cow yogurts, and the potassium content was lower. According to our dietary records, yogurts were adequately introduced into the habitual diets of the volunteers (unpublished data).

**Table 2.** Nutrient profile and fatty acid content (mean ± SD) of ewe’s and cow’s milk yogurts consumed in the study.

	Ewe’s milk yogurt (mean ±SD)		Cow’s milk yogurt
	Semi-skimmed	Whole	Whole
Protein (g/100 g)	5.9 ^a^ ± 0.5	5.8 ^a^ ± 0.5	3.2 ^b^ ± 0.04
Fat (g/100 g)	2.8 ^b^ ± 0.1	5.8 ^a^ ± 0.2	3.0 ^c^ ± 0.1
K (mg/kg)	1264.3 ^b^ ± 110.5	1243.0 ^b^ ± 90.6	1380.0 ^a^ ± 19.2
Ca (mg/kg)	2063.1 ^a^ ± 85.4	2012.2 ^a^ ± 110.9	1081.3 ^b^ ± 46.5
Mg (mg/kg)	178.1 ^a^ ± 29	178.8 ^a^ ± 23.7	84.3 ^b^ ± 7.7
TS (g/100 g)	14.1 ^b^ ± 0.40	16.7 ^a^ ± 0.6	11.2 ^c^ ± 0.1
SFA (g/100 g fat)	77. 85 ^a^ ± 2.03	79.59 ^a^ ± 1.69	73.87 ^b^ ± 2.30
MUFA (g/100 g fat)	19.38 ^b^ ± 2.08	17.96 ^b^ ± 1.72	23.35 ^a^ ± 1.75
PUFA (g/100 g fat)	2.77 ^a^ ± 0.40	2.45 ^a^ ± 0.43	2.78 ^a^ ± 0.60
SCFA (g/100 g fat)	20.26 ^a^ ± 2.40	21.17 ^a^ ± 1.96	11.31 ^b^ ± 1.86
MCFA (g/100 g fat)	20.99 ^ab^ ± 1.21	21.97 ^a^ ± 1.04	19.73 ^b^ ± 1.10
LCFA (g/100 g fat)	58.75 ^b^ ± 3.04	56.86 ^b^ ± 2.41	68.96 ^a^ ± 2.93
CLA (g/100 g fat)	0.27 ^a^ ± 0.12	0.24 ^a^ ± 0.12	0.26 ^a^ ± 0.08
n3 (g/100 g)	0.87 ^a^ ± 0.28	0.76 ^a^ ± 0.27	0.42 ^b^ ± 0.10
n6:n3	2.07 ^b^ ± 0.62	2.13 ^b^ ± 0.67	5.00 ^a^ ± 0.60
AI	7.37	7.49	6.68
Energy (kcal/100 g)	62.2	88.5	52.9

TS: total solids.

AI: atherogenic index = [C12:0 + (C14:0 × 4) + C16:0]/(Total unsaturated fatty acids).

Fatty acid analysis: semi-skimmed ewe’s yogurt (*n* = 9), whole ewe’s yogurt (*n* = 10), whole cow’s yogurt (*n* = 4).

Ewe’s milk yogurts: values followed by different superscript letters are different (p < 0.05).

**Figure 2.**
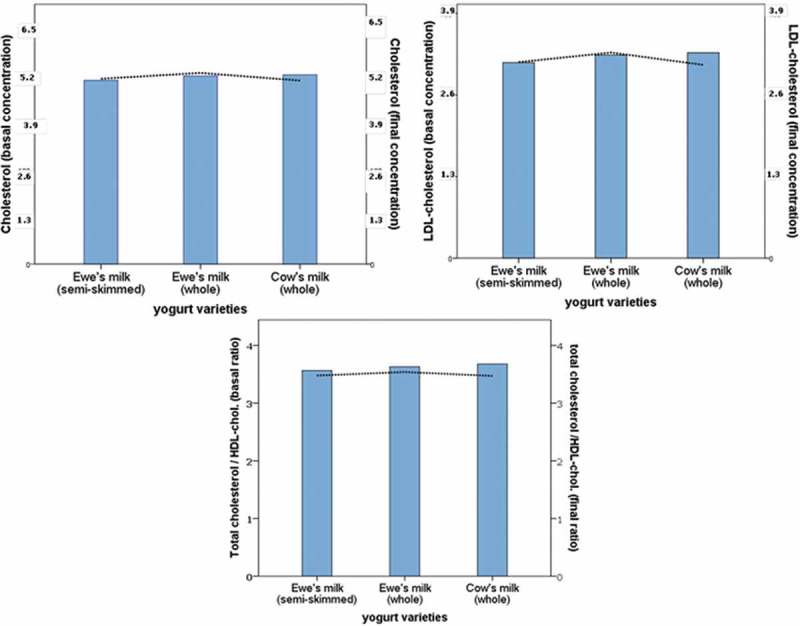
Baseline (bars and left axes) and final (dotted line and right axes) concentrations of serum total cholesterol, LDL-cholesterol (mmol/L) and, the total cholesterol/HDL-cholesterol ratio.

Table 3 lists the baseline and final concentrations of lipids and lipoproteins in each of the three intervention periods with semi-skimmed and whole ewe’s milk yogurts and whole cow’s milk yogurt. There were no differences in the serum concentrations of total cholesterol, HDL-cholesterol, LDL-cholesterol, and triglycerides of the volunteers at the begining of each of the three intervention periods (whole and semi-skimmed ewe’s milk, whole cow’s milk) (Figure 2), and there were no statistically significant differences after the consumption of any of the three types of yogurts, either in the whole sample or in the volunteers grouped according to their atherogenicity index (total cholesterol/HDL-cholesterol).

**Table 3.** Serum concentrations of cholesterol, HDL-cholesterol, LDL-cholesterol, and triglycerides (mmol/L, mean ± SD) and relative percentage changes of volunteers (*n* = 30) in the different intervention periods^a^.

	Whole cow’s milk yogurt	Whole ewe’s milk yogurt	Semi-skimmed ewe’s milk yogurt
**Total cholesterol**			
Baseline	5.23 ± 0.71	5.20 ± 0.73	5.08 ± 0.78
Final	5.07 ± 0.72	5.29 ± 0.76	5.12 ± 0.78
Rate of change	−0.03 ± 0.11	0.02 ± 0.09	0.01 ± 0.10
**HDL-cholesterol**			
Baseline	1.49 ± 0.36	1.51 ± 0.36	1.49 ± 0.35
Final	1.52 ± 0.38	1.54 ± 0.31	1.53 ± 0.31
Rate of change	0.03 ± 0.17	0.05 ± 0.12	0.04 ± 0.15
**LDL-cholesterol**			
Baseline	3.26 ± 0.67	3.20 ± 0.64	3.10 ± 0.67
Final	3.06 ± 0.60	3.26 ± 0.60	3.11 ± 0.70
Rate of change	−0.04 ± 0.16	0.02 ± 0.13	0.01 ± 0.18
**Triglycerides**			
Baseline	1.04 ± 0.37	1.09 ± 0.41	1.02 ± 0.40
Final	1.07 ± 0.39	1.08 ± 0.41	1.08 ± 0.43
Rate of change	0.06 ± 0.26	0.01 ± 0.27	0.09 ± 0.28

^a^No statistical differences were found in the baseline levels among the three phases of the study or after the consumption of any of the three types of yogurt.

**Figure 3.**
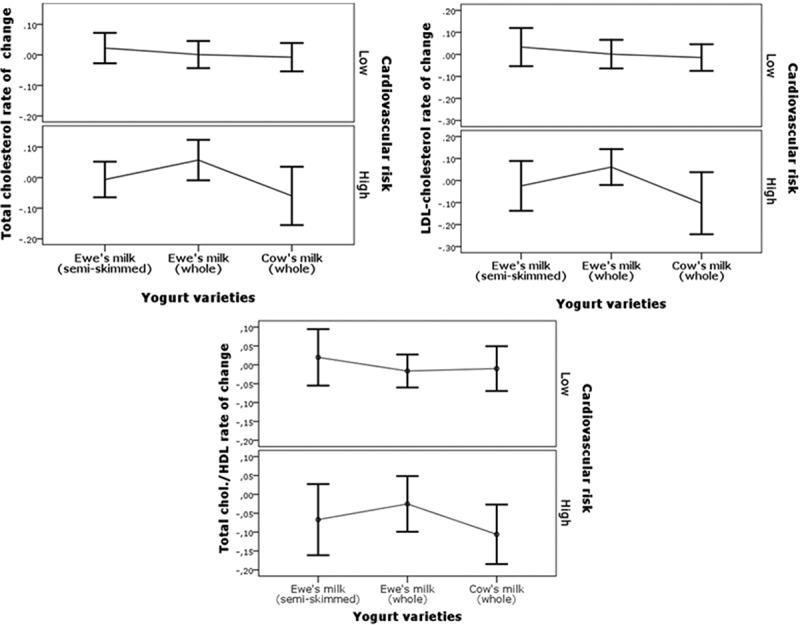
Relative percent changes in the concentrations of total cholesterol and LDL-cholesterol, and total cholesterol/HDL-cholesterol rate of changes after consumption of the different yogurt varieties. Values are expressed as means and 95% CI (error bars).

The relative percent changes in total cholesterol, HDL-cholesterol, LDL-cholesterol, and triglyceride concentrations after each of the three periods of yogurt consumption period are shown in Table 3. There were no significant differences in the variations obtained after the consumption of each of the three types of yogurt (Figure 3). Although the concentrations of serum cholesterol of the participants were normal or borderline high, the total sample was split into two groups of risk established according to the AI (total cholesterol/HDL-cholesterol), and the statistically significant differences observed at the beggining of the study between subjects at high and low risk in terms of the LDL-cholesterol, HDL-cholesterol, and triglyceride concentrations, were also observed in the responses to yogurt consumption with no effects due to the different types of yogurts (Table 4).

**Table 4.** Serum concentrations of cholesterol, HDL-cholesterol, LDL-cholesterol, and triglycerides (mmol/L, mean ± SD) and relative percentage changes of volunteers grouped according to the atherogenicity index (total cholesterol/HDL-cholesterol) (*n* = 10 at higher risk; *n* = 20 at lower risk) ^a^.

	Whole cow’s milk yogurt		Whole ewe’s milk yogurt		Semi-skimmed ewe’s milk yogurt	
	High risk	Low risk	High risk	Low risk	High risk	Low risk
**Total cholesterol**						
Baseline	5.58 ± 0.77	5.01 ± 0.58	5.62 ± 0.76	5.00 ± 0.64	5.71 ± 0.73	4.77 ± 0.59
Final	5.46 ± 0.63	4.88 ± 0.69	5.72 ± 0.59	5.06 ± 0.75	5.56 ± 0.64	4.91 ± 0.77
Rate of change	−0.03 ± 0.11	−0.02 ± 0.12	0.03 ± 0.11	0.02 ± 0.09	−0.02 ± 0.10	0.03 ± 0.10
**HDL-cholesterol**						
Baseline	1.39 ± 0.33	1.54 ± 0.37	1.39 ± 0.34	1.57 ± 0.36	1.37 ± 0.35	1.55 ± 0.34
Final	1.45 ± 0.28	1.56 ± 0.41	1.44 ± 0.31	1.59 ± 0.30	1.46 ± 0.36	1.56 ± 0.28
Rate of change	0.07 ± 0.17	0.01 ± 0.17	0.05 ± 0.12	0.05 ± 0.13	0.08 ± 0.16	0.02 ± 0.15
**LDL-cholesterol**						
Baseline	3.76 ± 0.63	3.00 ± 0.55	3.66 ± 0.51	2.97 ± 0.58	3.79 ± 0.46	2.76 ± 0.46
Final	3.46 ± 0.44	2.86 ± 0.58	3.71 ± 0.33	3.02 ± 0.58	3.50 ± 0.52	2.91 ± 0.70
Rate of change	−0.06 ± 0.14	−0.03 ± 0.17	0.03 ± 0.13	0.02 ± 0.13	−0.07 ± 0.13	0.06 ± 0.18
**Triglycerides**						
Baseline	1.11 ± 0.47	1.00 ± 0.32	1.24 ± 0.49	1.02 ± 0.36	1.21 ± 0.56	0.92 ± 0.33
Final	1.18 ± 0.42	1.02 ± 0.37	1.25 ± 0.49	0.98 ± 0.33	1.29 ± 0.48	0.97 ± 0.37
Rate of change	0.13 ± 0.18	0.02 ± 0.18	0.07 ± 0.36	−0.02 ± 0.20	0.11 ± 0.29	0.07 ± 0.27

^a^No statistical differences were found in the baseline levels among the three phases of the study or after the consumption of any of the three types of yogurt.

## Discussion

This study focused on yogurt from ewe’s milk (whole and semi-skimmed) and the effect of its consumption by apparently healthy average consumers on their blood lipid profile, compared with the consumption of cow’s yogurt. The volunteers included in this study had borderline high lipid levels (5.34 ± 0.71 mmol/L total cholesterol, 3.39 ± 0.72 mmol/L LDL-cholesterol, and 1.08 ± 0.55 mmol/L triglycerides). Cholesterol and LDL-cholesterol concentrations higher than 5.17 mmol/L and 3.36 mmol/L, respectively, are reported in 50% of the Spanish population [50]. The major finding was that, despite the different fat content, the consumption of ewe’s (whole) and cow’s (whole) yogurts, in quantities compatible with a varied diet, did not cause any significant changes in any of the traditional biomarkers of cardiovascular diseases (cholesterol, HDL-cholesterol, LDL-cholesterol, and triglycerides). No changes were observed in the lipid profile of volunteers when they were grouped according to the total cholesterol/HDL-cholesterol ratio (atherogenicity index), a lipoprotein ratio of clinical usefulness in cardiovascular prevention [51], regardless of the type of yogurt consumed.

Dairy products are major sources of SF; in the Spanish diet, they account for 20% of total SFA intake (6% and 2% of MUFA and PUFA, respectively) and most of the Spanish population surpasses the recommended SFA intake (96%) [12]. Dairy products and a high intake of SF have been linked to an increased risk of CVD [52]. However, recent findings have indicated that the link between CVD and SF may be less straightforward than originally thought [27]; although studies on the relationship between total dairy consumption and CVD risk markers have reported inconsistent results, the review of the literature could point to a potentital protective effect of full-fat milk, cheese, and yogurt, while the data pertaining to dairy fat are inconclusive [53]. Thus, there is a need for research on the effect of individual dairy foods on CVD risk [27,53,54] as well as on the differential effects of the fermented dairy products and the milk from which they are obtained, since there can be variations in their nutrient content [55,56]. Therefore, we focused on the effect of yogurt fermented with conventional startes cultures (*L. bulgaricus*, *S. thermophilus*) produced from cow’s milk (the most commonly consumed), for which there is little evidence indicating that it lowers serum lipids [27], and of yogurts prepared using ewe’s milk because of their different nutrient content and lipid profile. On the other hand, there are few studies on the effect of yogurt consumption on CVD risk factors [57–61] and, to our knowledge, only one involving ewe’s milk yogurt [57].

Although results from this study are difficult to compare with those reported in the literature because of many factors implicated in the experimental design (e.g. participant charateristicis, type and amount of yogurt consumed, length of the study), these are consistent with conclusions from other studies [58–61] that found no effects on cholesterol levels of normocholesterolaemic individuals when yogurt was consumed as a part of a habitual diet. However, the studies are not comparable in terms of the fat content of the cow’s milk yogurt used in these studies (low-fat [58,59]; skimmed yogurt [60]; whole milk yogurt [61]) or in the daily yogurt intake, which was higher in all of them (480 and 454 g/d, respectively, and higher in those by Rossouw et al. [61] and Thompson et al. [60], 1 L and 1.5 L) than the amounts consumed in the present study (250 g/d) or in the length of the studies (4 weeks [58,59], 3 weeks [60,61] vs 5 weeks in our study). Although not comparable because of the study design, a small reduction in plasma cholesterol concentration, but no change in the ratio of LDL- to HDL-cholesterol, was observed when cow’s dairy fat was replaced by sheep’s dairy fat in the dairy fat-rich diets of adults with serum cholesterol concentrations in the range of 4.78–7.76 mmol/L [57]. Cheese derived from sheep’s milk was given to volunteers (200 g/week, 10 weeks) to compare the effect of its consumption with that of a comercial cheese. Sofi et al. [62] found that although the protein and lipid content of the cheese made with sheep’s milk was higher, the plasma lipoprotein levels of the volunteers showed no differences. Another variable to be taken into account in studies of this type is the dietary intake. Although it was not controlled in this study, we instructed participants to maintain a mixed diet and to replace the yogurts with those provided in the study. On the other hand, this is a crossover study design to allow comparison within each individual, and the subjects were randomized into the three groups formed according to the intervention sequences, an aspect that contributes to the quality of the study [63], and the intervention period was almost twice that required to evaluate the effect of SF consumption on the cholesterol concentration [33] and higher than that in studies with similar aims [59–61].

Despite the fact that yogurt is a food commonly found in the Mediterranean diet, and even though it has been found to reduce the risk of CVD by about 30% [54], yogurt intake has hardly been studied in relation to those risk factors. However, it was part of a number of intervention studies involving dairy food products to assess the effect on the lipid profile of humans, as in the Predimed study, in which higher dairy consumption (yogurt, low-fat milk, and low-fat dairy products) was related to a decreased risk of developing metabolic syndrome [64]. Also in the context of the Mediterranean diet, in an older population at high CVD risk, low-fat yogurt consumption has been inversely associated with hypertriglyceridaemia, low HDL-cholesterol, and elevated fasting glucose [64].

Although cow’s milk is more frequently consumed, sheep’s milk products can also be found, especially in the Mediterranean countries. The major differences in the composition of milk and yogurts made from ewe’s and cow’s milk lie in the higher protein and fat content of the former, as well as higher amounts of vitamins, calcium, and phosphorus [15,65]. As indicated previously, ewe’s milk is richer in fat and SFA than cow’s milk, and a number of studies have shown that it offers a healthier nutritional profile than that of the cow, especially because of its higher content in short-chain FA (SCFA) from butyric to caproic acid, C4 to C10 (SCFA, see Table 2), associated with health benefits [66,67]. Besides SCFA, there are other beneficial lipids, such as the unsaturated FA, including MUFA (mainly oleic acid, which accounts for around 20%), as well as the PUFA (n-6 and n-3 FA), which includes CLA, and phosphor- and sphingolipids from the milk fat globule [68]. With respect to the fat content of dairy products, general dietary guidance to reduce total milk fat intake in order to decrease SFA consumption is currently considered inadequate, because that recommendation will also reduce the supply of bioactive lipid compounds and vitamins [67,69].

Nevertheless historically, total serum cholesterol and specifically LDL-cholesterol has served as a marker for the risk of CVD. It is recognized that whereas most of the SFA have a neutral effect on this serum LDL-cholesterol, several others, in particular C12, C14, and C16, which accounted for around 40% in milk fat, caused an increase in LDL-cholesterol. This led to the development of the AI (see Table 2) [20] which ranked foods based on their content of these three FA related to total unsaturated FA. However nowadays, it is well documented that those FA also cause an increase in HDL-cholesterol related to a lower risk of CVD. Therefore, the latest scientific evidence and meta-analyses indicate that the moderate consumption of whole dairy foods is not associated with an increased risk of CVD and is inversely associated with weight gain and the risk of obesity [68,70]

On the other hand, the SCFA are almost twofold higher in ewe’s yogurt than in cow’s yogurt (Table 2), which is related to its higher SFA content, although, as has been stated above, the SCFA have no effect on LDL-cholesterol concentration. However the palmitic acid (C16:0) content, which has been related to the AI and found to increase the risk of CVD, is much higher in cow’s milk fat than in ewe’s milk (34% vs. 28% respectively [data not shown]). This higher value of C16:0 and also of stearic acid (C18:0) in cow’s milk fat explains the significantly higher values of long-chain FA in this product. Although there were no significant differences in the PUFA or CLA content between yogurt samples, ewe’s milk yogurt provided almost twice the amount of n-3 alpha linolenic acid as cow’s milk yogurt and, hence, might be considered a much healthier product because it reduces the n-6:n-3 ratio, a commonly used biomarker of CVD [71].

The protein content in yogurts made from cow’s and ewe’s milk is also different, as it is related to whey proteins and to the profile of bioactive peptides with the beneficial effects of these products [72,73]. The hypocholesterolaemic effect of whey proteins has been observed in connection with casein in an intervention study [74]. Thus, the higher content of lipids, proteins, and calcium in ewe’s milk yogurts than in cow’s milk yogurt may enhance some benefits for health, without increasing serum lipid concentrations. The energy value of whole ewe’s milk yogurt may be a problem if the energy content of the diet is not controlled. However, from observational studies, it seems that a high yogurt consumption is associated with overall healthy diets [75]. This is acknowledged to be a confounding factor in the interpretation of the results, since part of the beneficial effect in CVD risk reduction might be attributable to the overall diet composition [54].

The present study shows that the intake of yogurt, from ewe’s and cow’s milk, at levels that the consumer might consider to be part of a varied diet, neither decreases nor increases plasma lipoprotein cholesterol levels in apparently healthy individuals with characteristics similar to those included in this study. However, ewe’s milk yogurt has no impact on the consumer lipid profile, is a major source of SCFA, calcium, and proteins, and has a reduced n6:n3 ratio. This may be favourable for certain population groups that could benefit from a product with a high density of said nutrients.
